# Can Long-Range PCR Be Used to Amplify Genetically Divergent Mitochondrial Genomes for Comparative Phylogenetics? A Case Study within Spiders (Arthropoda: Araneae)

**DOI:** 10.1371/journal.pone.0062404

**Published:** 2013-05-08

**Authors:** Andrew G. Briscoe, Sara Goodacre, Susan E. Masta, Martin I. Taylor, Miquel A. Arnedo, David Penney, John Kenny, Simon Creer

**Affiliations:** 1 Environment Centre Wales Building, Molecular Ecology and Fisheries Genetics Laboratory, School of Biological Sciences, College of Natural Sciences, Bangor University, Bangor, United Kingdom; 2 Institute of Genetics, Queens Medical Centre, University of Nottingham, Nottingham, United Kingdom; 3 Department of Biology, Portland State University, Portland, Oregon, United States of America; 4 Departament Biologia Animal, Universitat de Barcelona, Barcelona, Spain; 5 Faculty of Life Sciences, The University of Manchester, Manchester, United Kingdom; 6 Centre for Genomic Research, School of Biological Sciences, University of Liverpool, Liverpool, United Kingdom; Consiglio Nazionale delle Ricerche (CNR), Italy

## Abstract

The development of second generation sequencing technology has resulted in the rapid production of large volumes of sequence data for relatively little cost, thereby substantially increasing the quantity of data available for phylogenetic studies. Despite these technological advances, assembling longer sequences, such as that of entire mitochondrial genomes, has not been straightforward. Existing studies have been limited to using only incomplete or nominally intra-specific datasets resulting in a bottleneck between mitogenome amplification and downstream high-throughput sequencing. Here we assess the effectiveness of a wide range of targeted long-range PCR strategies, encapsulating single and dual fragment primer design approaches to provide full mitogenomic coverage within the Araneae (Spiders). Despite extensive rounds of optimisation, full mitochondrial genome PCR amplifications were stochastic in most taxa, although 454 Roche sequencing confirmed the successful amplification of 10 mitochondrial genomes out of the 33 trialled species. The low success rates of amplification using long-Range PCR highlights the difficulties in consistently obtaining genomic amplifications using currently available DNA polymerases optimised for large genomic amplifications and suggests that there may be opportunities for the use of alternative amplification methods.

## Introduction

Mitochondrial DNA markers are the cornerstones of contemporary molecular systematics and contribute greatly to our understanding of organellar evolution [1], [2]. However, robust phylogenetic reconstruction is often impeded by low sequence volume and number of comparative loci. Prior to the turn of the century, single gene partitions were commonly used to infer phylogenetic histories, but poor nodal support and discordance between phylogenies derived from separate markers clearly revealed that additional data are needed for robust phylogenetic reconstruction [3].

As more sequence data becomes available for elucidating the tree of life, large-scale sequencing efforts and interrogation of expressed sequence tag (EST) libraries or sequenced transcriptomes [4], [5] have begun to yield large numbers of nuclear markers that can be used for phylogenetic reconstruction [6]. However, true phylogenomic analyses are still not practical for the *de novo* construction of phylogenetic hypotheses for taxa without sequenced transcriptomes [7], [8]. A compromise between traditional phylogenetic methods and phylogenomics lies in the phylogenetic analysis of whole mitochondrial genomes [9]–[11]. However, the routine amplification of complete mitochondrial genomes from divergent taxa remains a significant hurdle to the widespread adoption of mitogenomic approaches.

The number of informative phylogenetic characters within the mitochondrial genome has been appreciated for some time [1] but most studies have limited themselves to only exploring parts of this information to resolve relationships at multiple levels [12]. The increase in whole mitochondrial genome datasets has provided new characters potentially allowing more robust phylogenies to be constructed. These markers, known as rare genomic changes (RGCs) have become increasingly popular for resolving complex phylogenetic relationships where traditional methods have produced ambiguous, unresolved results [12]. RGCs, defined as large-scale mutational changes occur much less frequently than base substitutions and have long been used in phylogenetics as supporting data embedded in DNA sequences. Examples of RGCs include changes in organelle gene order, gene duplications and genetic code variants [13]–[16].

Whilst the potential benefits of analysing whole mitochondrial genomes (including both coding regions and structural characters) in furthering our understanding of the mechanisms behind the evolution of organellar DNA are clear, all of the data produced to date have been of comparatively low volume. The lack of empirical data means that we still cannot accurately assess the utility of full mitogenomic sequences as a tool for resolving complex phylogenies in many taxa [17]. Advances in sequencing technology (e.g 454 Roche GSFLX series and Solexa/Illumina) are likely to resolve the sequencing throughput issue [18]. However, there are still clear limitations associated with the large-scale PCR amplification of divergent, interspecific, whole mitochondrial genomes. In spite of the recent revolution in sequencing technologies, current mitogenomic studies have been characterised by either analysing data from large, but incomplete mitochondrial genome fragments [10], low numbers of species [19] or by focusing on nominally intra-specific datasets [9]. In order to investigate the resolution potential, and associated problems with the amplification of large, interspecific mitogenomic data sets we focus here on spiders (Arthropoda: Araneae).

The Araneae are among the oldest and most diverse group of terrestrial organisms [20], [21], with a current diversity of more than 43,240 described species, placed in 111 families [22]. Spiders are an unequivocally ecologically important guild, being the dominant predators of insects in natural and managed ecosystems [23]. However, they have been relatively understudied from a higher-level molecular systematic perspective, and very little is known about inter-family relationships [24]. More recent attempts to resolve the phylogeny of the Araneae, have revealed significant topological incongruence between morphological and multiple loci phylogenies [25]. This makes the Araneae an ideal order to test the application of using mitochondrial phylogenomics to resolve complex relationships and better understand the evolutionary mechanisms underlying speciation and diversification. Furthermore, recent studies have shown repeated tRNA gene translocations [26], [27] in combination with an extensive fossil record can be utilised in resolving high-level relationships, via calibrated gene trees in the Araneae [28]–[31]. Here, we investigate the utility of direct long-range PCR amplification of whole mitochondrial spider genomes, using a large range of currently available long-range *Taq* polymerases. The PCR approach was chosen as it circumvents the need for (often unavailable) large starting biomass associated with the direct pelleting of mitochondria. Whilst mitochondrial genomes can be readily amplified in large numbers of small fragments, this is both costly and labour intensive. To this end, we adopted both single and dual fragment amplification approaches across the phylogenetic breadth of the order to assess the feasibility of both conserved and directed approaches for expedient whole mitochondrial DNA amplification.

## Materials and Methods

### Sample Collection and DNA Extraction

Spiders were obtained from across the United Kingdom and Gambia by the authors and members of the British Arachnological Society (BAS) and either ethanol preserved (70%–100%, stored at 4°C) or freshly frozen (stored at −80°C) directly from living individuals (Table 1). Samples of *Selenops annulatus*, *Deinopis* sp. and *Cithaeron praedonius* had been stored in 70% ethanol and were incorporated in order to maximise taxonomic coverage across the order. No specific permits were required for the described field studies and no specific permissions were required for these locations or activities. No locations were privately owned or protected and the field studies did not involve protected or endangered species. All four legs were removed from the left side of the thorax prior to DNA extraction. The rest of the body was stored in 100% ethanol for vouchering and subsequent identification purposes. Whole genomic DNA was extracted from a single femur of each species using the Qiagen DNeasy Blood & Tissue Kits (Qiagen).

**Table 1:** Taxonomic information, storage conditions and GenBank accession numbers for specimens.

Family	Genus	Specific epithet	Sample ID	Locality	COI	16 s	Sample storage
Agelenidae	*Malthonica*	*silvestris*	464_SC_AB	Kent, UK	JQ412460		Frozen
Amaurobiidae	*Amaurobius*	*similis*	483_SC_AB	Lancashire, UK		JQ406635	Frozen
Anyphaenidae	*Anyphaena*	*accentuata*	522_SC_AB	Kent, UK	JQ412439	JQ406633	Frozen
Araneidae	*Agalenatea*	*redii*	507_SC_AB	Kent, UK		JQ406637	Frozen
Araneidae	*Araneus*	*diadematus*	571_SC_AB	Dorset, UK	JQ412440	JQ406621	Frozen
Cithaeronidae	*Cithaeron*	*praedonius*	455_SC_AB	Kotu, Gambia	JQ412441		Ethanol
Clubionidae	*Clubiona*	*terrestris*	518_SC_AB	Kent, UK	JQ412442		Frozen
Corinnidae	*Messapus*	*martini*	453_SC_AB	Kerr Serign, Gambia		JQ406616	Ethanol
Corinnidae	*Phrurolithus*	*festivus*	503_SC_AB	Kent, UK		JQ406632	Ethanol
Ctenidae	*Anahita*	sp.	460_SC_AB	Kotu, Gambia		JQ406614	Ethanol
Cybaeidae	*Argyroneta*	*aquatica*	581_SC_AB	Dorset, UK		JQ406617	Frozen
Deinopidae	*Deinopis*	sp.	451_SC_AB	Gunjur, Gambia	JQ412443		Ethanol
Dictynidae	*Dictyna*	*latens*	499_SC_AB	Kent, UK	JQ412444	JQ406629	Ethanol
Dysderidae	*Dysdera*	*erythrina*	479_SC_AB	Kent, UK	JQ412445	JQ406627	Ethanol
Eresidae	*Stegodyphus*	sp.	454_SC_AB	Bijilo, Gambia	JQ412459		Ethanol
Gnaphosidae	*Zelotes*	*apricorum*	462_SC_AB	Kent, UK	JQ412463		Frozen
Idiopidae	*Gorgyrella*	sp.	448_SC_AB	UK Pet Trade	JQ412447		Frozen
Linyphiidae	Unknown	Unknown	559_SC_AB	Gwynedd, UK	JQ412448	JQ406636	Frozen
Lycosidae	*Pardosa*	*nigriceps*	477_SC_AB	Kent, UK	JQ412452	JQ406631	Ethanol
Philodromidae	*Philodromus*	*dispar*	517_SC_AB	Kent, UK	JQ412453	JQ406634	Frozen
Pisauridae	*Pisaura*	*mirabilis*	502_SC_AB	Kent, UK	JQ412454	JQ406630	Ethanol
Pholcidae	*Pholcus*	*phalangioides*	484_SC_AB	Lancashire, UK		JQ406625	Frozen
Salticidae	*Salticus*	*scenicus*	423_SC_AB	Gwynedd, UK	JQ412456	JQ406628	Ethanol
Segestriidae	*Segestria*	*senoculata*	583_SC_AB	Gwynedd, UK	JQ412457	JQ406615	Ethanol
Selenopidae	*Selenops*	*annulatus*	449_SC_AB	Kotu, Gambia	JQ412458		Ethanol
Sparassidae	*Micrommata*	*virescens*	461_SC_AB	Kent, UK	JQ412451	JQ406618	Frozen
Tetragnathidae	*Meta*	*menardi*	481_SC_AB	Gwynedd, UK	JQ412449	JQ406620	Frozen
Theraphosidae	*Eupalaestrus*	*campestratus*	446_SC_AB	UK Pet Trade	JQ412446	JQ406626	Frozen
Theraphosidae	*Grammostola*	*rosea*	445_SC_AB	UK Pet Trade		JQ406624	Frozen
Theraphosidae	*Psalmopoeus*	*cambridgei*	447_SC_AB	UK Pet Trade	JQ412455	JW406623	Frozen
Theridiosomatidae	*Theridiosoma*	*gemmosum*	489_SC_AB	Glamorgan, UK	JQ412461		Frozen
Thomisidae	*Xysticus*	*audax*	521_SC_AB	Kent, UK	JQ412462	JQ406622	Frozen
Uloboridae	*Miagrammopes*	sp.	452_SC_AB	Bijilo, Gambia	JQ412450	JQ406619	Ethanol

Sample storage indicates the methods in which the specimens were preserved on collection; either freshly frozen at −80°C (frozen) or stored in 70–100% ethanol at 4°C.

### Primer Anchoring Strategy

In order to identify an efficient approach for the amplification of whole mitochondrial genomes we adopted two long-range PCR strategies to amplify the complete mitogenome in one or two large fragments [32]. DNA from two anchoring regions, a ca. 650 b.p. region of the Cytochrome Oxidase I (COI) and a 450 b.p. region spanning the large ribosomal subunit (16****s rRNA), were amplified for 33 taxa. Both amplifications were performed in 25 µl reactions using the primer combinations of CHELF1 (5′- TACTCTACTAATCATAAAGACATTGG) and CHELR2 (5′-GGATGGCCAAAAAATCAAAATAAATG) (COI) [33] and primers LR-N-13398 (5′- CGCCTGTTTAACAAAAACAT) and LR-J-12887 (5′- CCGGTCTGAACTCAGATCACGT) (16****s) [25]. PCR reactions comprised 1× PCR buffer, 1.5 mM MgCl_2_, 0.4 µM of each primer, 0.625 units ThermoPrime *Taq* DNA polymerase (Thermo Scientific) and 1 µl template DNA and thermocycling was performed using a DNA engine (Tetrad 2) Peltier Thermal Cycler (BIORAD). Cycling conditions were 60 seconds at 94°C, 5 cycles of 60 seconds at 94°C, 90 seconds at 45°C and 90 seconds at 72°C followed by 35 cycles of 60 seconds at 94°C, 90 seconds at 50°C and 90 seconds at 72°C [33]. Amplification success was checked using a 1% agarose gel stained with Ethidium Bromide (EtBr). Successful amplifications were cleaned with 1****U shrimp alkaline phosphatase (Promega) to dephosphorylate residual deoxynucleotides and 0.5 U Exonuclease I (Promega) to degrade excess primers [34] and subsequently sequenced bidirectionally (Macrogen Inc, Seoul, Korea) using the same primers as for amplification.

### Long-Range Primer Design and PCR

**Figure 1:**
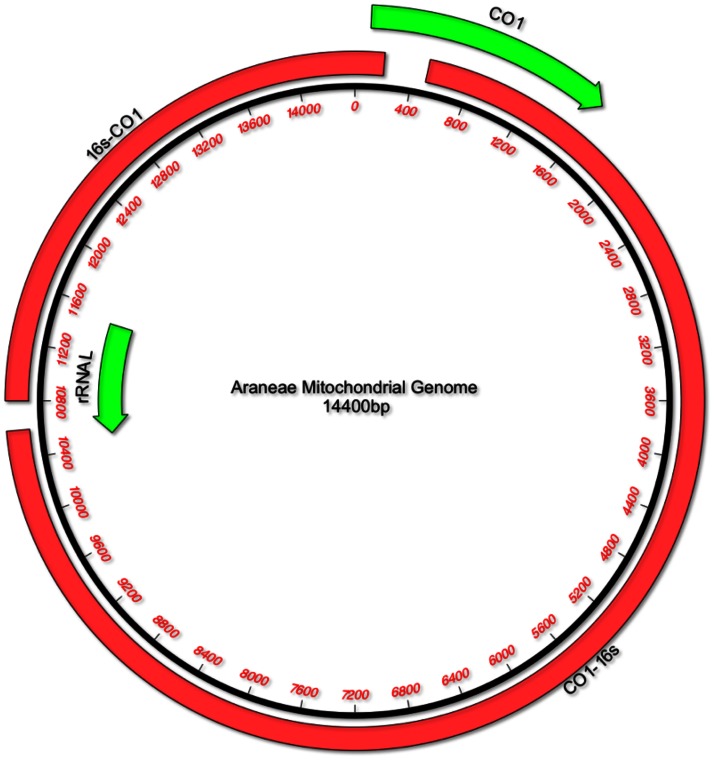
Araneae mitochondrial genome displaying anchoring regions. Spider mitochondrial genome highlighting the genes from which the two fragment long-range PCR strategy was designed (green arrows – direction indicates gene sequence 5′–3′). Solid red bars show the two long-range PCR products and approximate length.

Following the Sanger sequencing of the COI and 16****s anchoring regions, chromatographs were checked and sequences were manually edited where necessary, using CodonCode Aligner (v. 2.0.6, Codon Code Corporation), prior to alignment using Clustal W [35]. The initial aim of this process was to amplify regions of sufficient length, on opposite sides of the mitochondrial genome, from which ‘universal’ primers could be designed [26], [36], [37]. However, no conserved regions were found that would facilitate the design of long-range primers that could be used to amplify homologous loci from multiple families. long-range primers were subsequently designed for individual taxa in order to amplify the entire mitochondrial genome in one or two large fragments that overlapped with the conserved COI and/or 16****s ribosomal subunit using the Primer 3 software [38]. Default values were used with the exception of length (22–30****bp), primer T_m_ (57.0–70.0°C) and GC content (40–60%) which followed consensus recommendations from the *Taq* manufacturers (Primer sequences available on DRYAD entry doi:10.5061/dryad.8dd3n). For the single fragment protocol, primers were designed within the COI sequences, with the light strand primer situated downstream of the heavy strand primer, thus taking advantage of the circular nature of the genome. For the dual fragment protocols, two sets of primers were designed for each taxon, to bridge the gaps between the COI and 16****s regions (Figure 1).

The PCRs were performed initially in 50 µl volumes using multiple polymerases recommended for long-range PCR amplification, including: Clontech Advantage 2, Clontech Advantage Titanium, Clontech Advantage HD (Takara Biotech), NEB LongAmp (NEB), GeneAmp XL (Applied Biosystems) & Expand Long Range PCR (Roche), using the protocols and cycling conditions recommended by the manufacturers. All DNA polymerases were initially tested on five phylogenetically disparate samples prior to using the most successful ones on the remaining samples. Successfully amplified PCR products were electrophoresed and subsequently purified using Qiagen QiaQuick Gel extraction kits (Qiagen) or Origene Rapid PCR Purification System (Origene), dependent on fragment size. Following long-range PCR amplification, primers were assigned to groups based on amplification success (good, stochastic or no amplification) and subsequently tested for significant differences in primer length, GC content and T_m_ using a Mann-Whitney U test in the statistics package SPSS [39].

### Amplicon Shearing, Library Construction and 454 Roche Sequencing

Amplicons from eleven purified samples (*Meta menardi*, *Xysticus audax*, *Psalmopoeus cambridgei*, a Gorgyrella sp., Eupalaestrus campestratus, a Linyphiidae sp., *Zelotes apricorum*, *Malthonica silvestris*, *Araneus diadematus*, *Pisaura mirabilis* & *Dysdera erythrina*) were fragmented using a Covaris DNA shearer (Covaris) at 10% duty cycle, intensity: 4 with 200 cycles per burst for 65 seconds. Following quantification using a Qubit 2.0 fluorometer (Invitrogen), MID barcoded adapted sequencing libraries were constructed using the NEBNext DNA sample prep master mix set in accordance with manufacturer’s instructions (New England Biolabs), pooled in equimolar concentrations and sequenced at low putative coverage on 1/16^th^ of a 454 Roche GSFLX Titanium platform (Centre for Genomic Research, University of Liverpool). Please note that the sequencing step here was intended as a quality control measure and not intended to sequence to a depth required for full mitochondrial genome assembly (sequence quality data available on DRYAD entry doi:10.5061/dryad.8dd3n). The 454 Roche sequences were assembled using GS *De Novo* Assembler software (Roche). Following trials with Roche’s GS *De Novo* software, MIRA (B. Chevreux) and CLC Genomics Workbench (CLC bio, Aarhus, Denmark), no substantial differences in number of contigs or length were present using any of the approaches. Therefore, in accordance with the proven functionality of the 454 Roche software, all sequences were assembled using the GS *De Novo* program. Following assembly, the resulting contigs where compared to existing Araneae mitochondrial DNA sequences within GenBank via BLAST [40] in order to investigate sequence homology (Genbank accession numbers: AY309258, AY309259, AY452691, NC_005942, NC_010777, NC010780).

## Results

### Anchoring Regions

Of the 33 species used, 27 and 24 taxa were successfully amplified for the COI and the 16****s regions respectively, yielding anchoring points for a total of 26 families (Table 1) (GenBank accession numbers: COI - JQ412439 - JQ412463 and 16****s - JQ406614–JQ406637).

### Mitochondrial Genome PCR Amplifications

Initially, five specimens, covering a broad taxon range, were used to test the performance of the *Taq* polymerases for single fragment mitochondrial genome amplification (Table 2). Following repetition of the *Taq* testing when opting for a dual fragment approach, it was found that NEB LongAmp was equally as effective as Clontech Advantage 2 *Taq* polymerase. Subsequently, complete mitochondrial genomes were successfully amplified in c. 7–9 kb or c. 15 kb fragments using Clontech Advantage 2 (single fragment) or NEB LongAmp (dual fragment) *Taq* polymerases. The probability of obtaining successful amplifications of complete mitochondrial genomes utilising either the 1 or 2 fragment amplification protocol was not demonstrably different (based upon presence/absence of bands), suggesting that the limiting factor in the amplification of the genomes was not necessarily the size of the target fragment. In some two-fragment amplifications (Samples *Pardosa nigriceps*, *Dysdera erythrina*, *Pisaura mirabilis*, *Phrurolithus festivus* & *Anyphaena accentuata*) the COI to 16****s region amplified consistently more often than the reverse, highlighting potential inhibition to amplification within the target sequence. However, of the initial 33 samples trialled, from which a subset of 22 (based on sequence availability and prior PCR amplification success) were used for long-range PCR, only 11 mitochondrial genomes could be amplified robustly and with a guaranteed level of reproducibility (*Meta menardi, Xysticus audax, Psalmopoeus cambridgei,* a *Gorgyrella* sp., *Eupalaestrus campestratus,* a Linyphiidae sp., *Zelotes apricorum, Malthonica silvestris, Araneus diadematus, Pisaura mirabilis* & *Dysdera erythrina*). The NEB LongAmp polymerase (NEB) amplified genomes in two fragments with greater reproducibility whilst Clontech Advantage 2 (Takara Biotech) amplified single fragment genomes with greater consistency. Statistical analysis of the primer properties (length, GC content and T_m_) yielded no significant differences between any combination of primers that amplified repeatedly, intermittently, or those that consistently failed to amplify mitochondrial genome fragments.

**Table 2:** Enzymatic information for successful long-range amplifications.

Sample reference	Clontech		NEB LongAmp	Expand Long Range
	Advantage2	AdvantageLA		
448_SC_AB	Stochastic	No amplification	No amplification	Good
462_SC_AB	Good	No amplification	Weak	No amplification
464_SC_AB	Good	No amplification	No amplification	No amplification
446_SC_AB	No amplification*	No amplification	No amplification*	No amplification
451_SC_AB	Stochastic	Stochastic	No amplification	Stochastic

Clontech Advantage Titanium and Roche GeneAmp XL are not shown but failed to amplify any samples.

* Indicates good amplification from a dual fragment PCR approach. The term Good indicates robust and consistent amplification. The term Stochastic indicates a non-consistently reproducible amplification.

### 454 Roche Sequencing

The sequencing of the 11 amplicon libraries provided a total of 27,892 tagged reads with an average length of approximately 278 bases (Table 3). Reads from sample 464_SC_AB (*Malthonica silvestris*) were unable to be recovered by the MID identifying software. *De novo* read assembly was unable to construct complete mitochondrial genomes but provided an average of an estimated 65% sequence assembly (assuming a ca. 15 kb mitogenome target; contigs and sequences available on DRYAD entry doi:10.5061/dryad.8dd3n).

**Table 3:** Sequencing information following 454 GS FLX run and subsequent GS *De Novo* Assembler contig assembly.

Species Name	Sample Reference	Number of Reads	Number of Contigs	Average Contig Length	Roche MID Identifier	Longest Contig Length
*Araneus diadematus*	571_SC_AB	1,313	20	340	1	1,058
*Psalmopoeus cambridgei*	447_SC_AB	1,689	17	944	2	4,483
*Eupalaestrus campestratus*	446_SC_AB	380	5	849	3	1,916
*Gorgyrella* sp.	448_SC_SB	6,203	49	665	4	2,849
*Xysticus audax*	521_SC_AB	4,887	35	423	5	2,257
*Pisaura mirabilis*	502_SC_AB	1,192	14	505	6	872
*Dysdera erythrina*	479_SC_AB	1,554	16	733	7	1,523
*Linyphiidae* sp.	559_SC_AB	503	12	540	8	1,310
*Zelotes apricorum*	462_SC_AB	9,081	26	619	9	6,434
*Meta menardi*	481_SC_AB	1,090	18	532	11	1,452

Number of reads per sample represents only those from which the indicator MID sequence was recovered. Number of contigs represents only those formed with a length greater than 100 bases. Average contig length refers only to contigs over 100 bases in length. Longest contig length shows the largest single contig assembled.

In light of the likelihood of tRNA gene rearrangements and because only 6 mitochondrial genomes were available on Genbank at the time of analysis, a highly dissimilar BLAST search was performed on the nucleotide collection database. Of the 10 samples, all contigs created through short-read assembly had maximum identification values ranging from 78–100% Max ID for spider mitochondrial DNA, either through an unrestricted blast search or with searches focused on Araneae accessions. Nucleotide sequences for all reads were deposited in the GenBank short-read archive (SRA051390.1).

## Discussion

We were able to amplify most of the target genes (COI and 16****s) across our taxon range, but were unable to identify or develop degenerate primers for any other potential anchoring region within the spider mitochondrial genome following sliding window analyses of all regions most commonly used in spider phylogenetics. Sliding window analysis was performed on all 13 protein coding genes and both ribosomal RNAs (rRNAs) of the six previously published spider mitochondrial genomes using the *Drosophila* Polymorphism database, SNP Graphics (http://dpdb.uab.es/dpdb/diversity.asp) (sliding window plots available on DRYAD entry doi:10.5061/dryad.8dd3n). This most likely highlights the high level of mitochondrial genetic diversity that can be found within the spiders along with the absence of universal primers available for no more than a handful of protein coding genes, resulting in few reference sequences available for comparison for large portions of the Araneae mitogenome [41].

Predictably, the 454 Roche low coverage sequencing step resulted in a wide variation of read coverage per amplicon pool, most likely due to differences in MID primer tag design and read recovery. Nevertheless, even highly covered mitochondrial genomes did not result in a full assembly, suggesting further optimisation may be required in either the shearing or bioinformatic steps of mitogenome assembly [42]. The BLAST search of the assembled contigs showed that spider mitochondrial genomes had indeed been amplified and that we had not inadvertently amplified nuclear DNA or DNA from a contaminating source such as the *Wolbachia* bacterium [43]. The need to use searches focused on Araneae accessions, in order to get positive matches, most likely highlights the genetic divergence between the trialled taxa and the complete mitogenomic data currently available for 6 out of the 112 described families of spiders.

Nevertheless, even following an extensive campaign of PCR strategies and optimization, using a variety of long range *Taqs*, we could not consistently amplify approximately two thirds of the species, a substantial proportion of our sample taxa. We have no reason to attribute PCR failure to degraded DNA resulting from sample storage conditions, since all specimens were preserved directly from living organisms using tried and tested preservation media. Moreover, of the three samples stored in 70% EtOH, although sample *Selenops annulatus* did not amplify, samples *Deinopis* sp. and *Cithaeron praedonius* could be amplified, albeit inconsistently, suggesting that 70% EtOH preservation over ca. three years may be sufficient to preserve mitogenome integrity for PCR amplification. However, we could not rule out the possibility that sub-optimal storage conditions can adversely affect the availability of suitable templates for long-range PCR. It is also unlikely that the primers affected long-range PCR success rate, since comparative analysis between the primer sets revealed no discernable statistical differences in physical or chemical properties (i.e. GC content, length, T_m_). The arachnid mitochondrial genome, as for most animal mitochondrial genomes, is a small extra-chromosomal genome comprising 37 genes including 22 coding for transfer RNAs (tRNAs), 13 coding for proteins and 2 coding for ribosomal RNAs (rRNAs) [1]. As well as the 37 coding genes, mitochondrial genomes also contain a small, approximately 1–2 kb, non-coding control region, named due to its perceived role in controlling the transcription and replication of the mtDNA molecule [44]. However, the protein coding gene arrangement of spiders is highly conserved and shared amongst many other Chelicerates and so is not likely to cause differences in amplification success. Previously published Araneae mitochondrial genomes are considered to be very A/T rich (64–76%), reflecting the low G/C content of the mitochondrial genomes of other arthropod orders [45]. In spite of the low G/C content of the DNA, no definitive reason is forthcoming for the failure to obtain more consistent long PCR amplifications from our target mitochondrial genomes. Whilst the larger region (COI-16****s) of the dual fragment approach amplified successfully more often than the shorter 16****s-COI counterpart, the G/C content of both fragments is comparable (approximate difference of 2.6% averaged across all currently published Araneae mitochondrial genomes) with no significant differences between the highest and lowest values of the comprising genes within each fragment. However the lesser-amplified small fragment (16****s-COI) does contain the control region, also known as the A+T rich region due to its low G/C content, which has been known to inhibit DNA polymerases in insects and platyhelminths [46]. However, enzyme inhibition is more likely to be caused by tandem repeats [47] present in and around the control region as comparative analysis of previously published arachnid mitochondrial genomes show other genes to also have lower G/C content.

Our results provide evidence for the successful amplification of several whole mitochondrial genomes, in one or two long fragments overlapping the COI and/or 16****s regions, using a variety of commercially available DNA polymerases optimised for large genomic amplifications. However, we have also highlighted the difficulty in obtaining amplifications with a guaranteed level of reproducibility, using multiple currently available polymerases and thus highlighted potential limitations to the feasibility of mitogenomic studies featuring large numbers of independently amplified taxa using single, or dual fragment approaches. Recent studies utilising mitogenomics to resolve phylogenetic incongruences have avoided this issue by focusing on the production of datasets comprising either incomplete genome sequences [10] or that are nominally intra-specific [9]. Whilst these data still provide a large number of informative phylogenetic characters, the lack of the whole mitochondrial genome precludes the acquisition of maximum phylogenetic resolution, including RGCs, from the mtDNA genome [48]–[50]. However, the importance of recovering complete mitogenomic sequences in order to more accurately reconstruct phylogenetic relationships has long been understood and so remains an important avenue of exploration in contemporary phylogenetics [51]. Although we present data on a single order (Araneae – Spiders) we believe that the results highlight the limitations to the feasibility of generating diverse, interspecific complete mitochondrial genome data sets from long-range PCR amplifications, in terms of both cost and efficiency. Whilst many studies have successfully used a multitude of PCR amplifications in order to generate mitochondrial genomes [48], [52], [53], this is both labour and time intensive. This highlights the potential need to utilise alternative, non-PCR based methods that are able to amplify complete mitochondrial genomes both quickly and cost effectively. Direct recovery of organellar genomes and mitochondrial gene partitions from whole shotgun genome sequencing and EST libraries is possible [54], [55], but expedient mechanisms to isolate only the mtDNA locus are needed to optimise organellar coverage for large numbers of taxa. While methods such as Rolling Circle Amplification (RCA) [56] have been trialled successfully on a limited number of species, further investigations across a range of taxa will be desirable to investigate their full potential to create divergent, multi-taxon datasets for comparative mitogenomics. Such methods hold advantages over PCR-based strategies due to the non-specific nature of the amplification process. By using random hexamers, as opposed to synthesising bespoke taxon-specific oligonucleotides, it is possible to avoid the amplification failure shown by this study whilst allowing for the creation of large, diverse mitochondrial genome datasets from low amounts of starting material.

### Data Accessibility

DNA sequences: Genbank accessions JQ412439 - JQ412463**,** JQ406614–JQ406637 and SRA051390.1.

Primer sequences, Contigs & Sliding window analysis: DRYAD entry doi:10.5061/dryad.8dd3n.
